# Graphene-enhanced Raman spectroscopy of thymine adsorbed on single-layer graphene

**DOI:** 10.1186/s11671-015-0869-4

**Published:** 2015-04-02

**Authors:** Olena Fesenko, Galyna Dovbeshko, Andrej Dementjev, Renata Karpicz, Tommi Kaplas, Yuri Svirko

**Affiliations:** Institute of Physics, National Academy of Sciences of Ukraine, 46 Nauki Ave., Kyiv, 03680 Ukraine; Center for Physical Sciences and Technology, Institute of Physics, A. Goštauto 11, Vilnius, LT-01108 Lithuania; Institute of Photonics, University of Eastern Finland, Yliopistokatu 7, Joensuu, FI-80101 Finland

**Keywords:** Single-layer graphene, Thymine, Surface-enhanced Raman spectroscopy (SERS), Graphene-enhanced Raman scattering (GERS), Coherent anti-Stokes Raman scattering (CARS), Graphene-enhanced coherent anti-Stokes Raman scattering (GECARS)

## Abstract

Graphene-enhanced Raman scattering (GERS) spectra and coherent anti-Stokes Raman scattering (CARS) of thymine molecules adsorbed on a single-layer graphene were studied. The enhancement factor was shown to depend on the molecular groups of thymine. In the GERS spectra of thymine, the main bands are shifted with respect to those for molecules adsorbed on a glass surface, indicating charge transfer for thymine on graphene. The probable mechanism of the GERS enhancement is discussed. CARS spectra are in accord with the GERS results, which indicates similar benefit from the chemical enhancement.

## Background

Surface-enhanced Raman spectroscopy (SERS) has become an efficient technique that enables detection and study of an extremely small amount of biochemical materials and single-molecule detection [1-4]. SERS is based on the enhancement of the local optical field by several orders of magnitude in the vicinity of a rough metal surface or metal island film due to excitation of the collective oscillations of conduction electrons at the metal surface (surface plasmons). However, investigation of the biological and biochemical species often requires substrates of higher chemical inertness. Such substrates can be based in particular on carbon allotropes, e.g., on graphene or carbon nanotubes. But in graphitic materials, the surface plasmon resonance is found in the THz range [5,6], i.e., plasmon-based local field enhancement can hardly be employed in optical spectroscopy with such carbon-based substrates. Nevertheless, it has been recently demonstrated [7-10] that the Raman signal of molecules deposited on graphene and graphene oxides is enhanced by several orders of magnitude, which is likely to be caused by the so-called chemical mechanism [11], i.e., chemical interaction of deposited molecules and carbon atoms of the substrate. This phenomenon is called graphene-enhanced Raman scattering (GERS) [12] and may become important for spectroscopy of certain biological and biochemical species. In particular, we have recently reported the enhancement for Raman and coherent anti-Stokes Raman scattering of thymine adsorbed on graphene oxide [10].

In the present paper, we report on a comparative study of the surface-enhanced Raman scattering and surface-enhanced coherent anti-Stokes Raman scattering for thymine (Thy) adsorbed on graphene layers.

### Samples

In the Raman and coherent anti-Stokes Raman scattering (CARS) measurements, we use aqueous 1 mg/ml and 10 μg/ml solutions of commercially available thymine (Sigma-Aldrich, St. Louis, MO, USA). The samples for optical experiments were prepared by depositing a drop of Thy solution on graphene-on-silica or glass substrates, respectively. The average surface density of Thy after water evaporation was either 200 ng/cm^2^ or 20 μg/cm^2^.

The graphene-on-silica substrates were fabricated by depositing a graphene sheet on fused silica. Single-layer graphene was prepared by using chemical vapor deposition (CVD) of graphene on a copper foil described elsewhere [13,14]. Before the start of the graphitization process, the copper substrate was annealed for 1 h at a temperature of 1,000°C in 15-mbar hydrogen atmosphere. After the annealing, the CVD chamber was pumped down and filled with 1:1 H_2_:CH_4_ gas mixture for 10 min (15 mbar). Because of the self-limiting graphene growth on a copper substrate, almost single-layer graphene [15] was deposited on both surfaces of the copper foil. The templated graphene growth was suppressed by short hydrogen etching at a temperature of 1,000°C and pressure of 50 mbar [16]. After the etching, the CVD chamber was cooled down to room temperature in hydrogen atmosphere (15 mbar). The graphene deposited on the backside of the copper was etched away in harsh oxygen plasma (100 W/20 sccm/2 min).

The graphene sheet was spin coated by a 1-μm-thick polymethyl methacrylate (PMMA) layer, and then the copper foil was removed by wet etching in FeCl_3_. The remaining PMMA/graphene stack was rinsed in deionized water for 30 min and then placed on a silica substrate in such a way that the graphene was facing the silica. In order to relax internal stress in the stack, another PMMA layer was deposited on top of the existing PMMA [17]. After removal of both the PMMA layers by acetone, the obtained graphene-on-silica substrate was rinsed in isopropanol and water. The procedure described above is scalable, i.e., one can add one or more graphene sheets on the top of the first one using the same technique.

## Methods

### Raman measurements

The Raman spectra of the bare and Thy-adsorbed graphene layers were obtained using a Renishaw inVia Raman microscope (Renishaw plc, Wotton-under-Edge, UK) at an excitation wavelength of 633 nm and spot size of 1 μm. All Raman measurements were performed at room temperature. The WiRE 3.4 software (Renishaw) was used for Raman data acquisition and data analysis. The Si Raman band centered at 520 cm^−1^ was used as the reference.

### CARS measurements

The experimental setup was based on a homemade CARS microscope equipped with a compact laser source (EKSPLA Ltd., Vilnius, Lithuania) capable of providing pump and Stokes pulses with energies up to 10 nJ (see [10,18] for more details). The excitation beams were focused on the sample with an oil immersion objective (Plan Apochromat, ×60, NA 1.42, Olympus Corporation, Tokyo, Japan). The CARS mapping of the Thy-adsorbed graphene-on-silica substrate and glass was performed with a spatial resolution of approximately 0.5 and 1.0 μm in the lateral and normal (*z*) directions, respectively. The CARS images of the sample surface were composed of 250 × 250 pixels obtained with a 2-ms pixel dwell time using a piezo scanning system (Physik Instrumente GmbH & Co., Karlsruhe, Germany). CARS spectra were recorded with a scanning rate of 5 cm^−1^ s^−1^ in the frequency ranges of 1,200 to 1,700 cm^−1^ and 2,500 to 3,400 cm^−1^ with a resolution of about 8 cm^−1^.

## Results and discussion

### Raman spectra of the stacked graphene sheets

**Figure 1:**
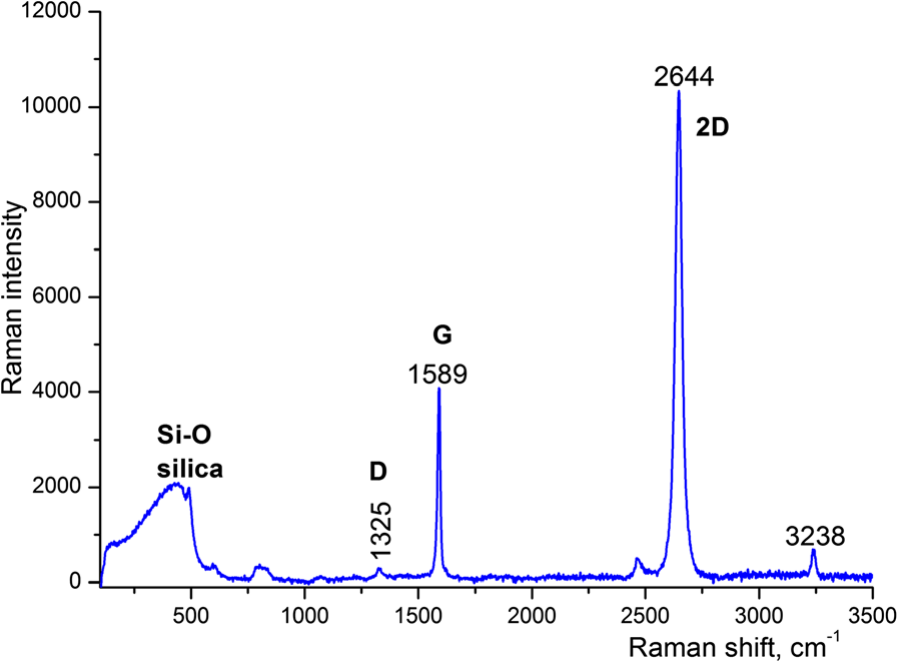
**Raman spectra of single-layer graphene collected at 633 nm.**

**Figure 2:**
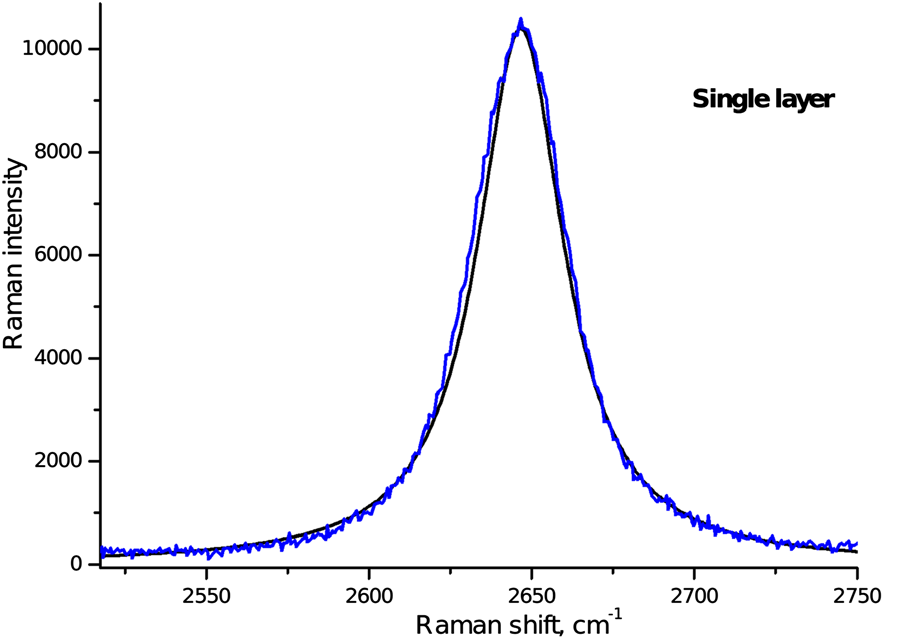
**2D Raman peak of the single-layer graphene obtained at an excitation wavelength of 633 nm.** Experimental band (blue) and Lorentzian peak (black).

Raman spectra of the fabricated graphene samples (see Figure 1) dominate G and 2D bands. The shape and position of the 2D peak indicate that the samples consist of one graphene layer (Figure 2). A relatively weak D peak situated in the vicinity of 1,325 cm^−1^ indicates the presence of defects in the graphene [19-22]. The ratio of the D and G peaks can be employed for quantitative characterization of the crystallinity. Specifically, if we assume that there is no amorphous and/or carbon and sp^3^-bonded carbon in the samples, the size of the graphene crystallites *L*_*a*_ can be estimated according to Tuinstra-Koening (TK) [22], which yields *L*_*a*_ ~ *I* (D)/*I* (G) where *I* (D) and *I* (G) are intensities of the G and D peaks in the Raman spectrum. In our case, the ratio *L*_*a*_ does not exceed 0.1 for single-layer graphene, indicating a good crystallinity of the fabricated graphene.

The position *ω*_G_ of the G peak can be employed to estimate the number of graphene layers *n* on the silica substrate by using the following equation [23]: *ω*_G_ = 1,581.6 + 11/(1 + *n*^1.6^).

The obtained experimental value of *ω*_G_ = 1,589 cm^−1^ corresponds to *n* ~ 1. It should be mentioned that this layer had defects that is proved by the presence of the weak D peak situated in the vicinity of 1,325 cm^−1^. The ratio *I* (G)/*I* (2D), which also can be used to estimate the number of graphene layers, does not exceed 0.4, indicating the presence of one to two layers of graphene. These results correspond well to SEM images of graphene-on-silica substrates that show gaps and defects in the graphene sheets.

The Raman spectrum of the bare graphene-on-silica substrate includes a pronounced band at 450 cm^−1^ which originated from symmetric stretching vibrations of neighboring Si-O bonds [24,25]. The band near 800 cm^−1^ is also associated with symmetric stretching vibrations of oxygen atoms, which involve also a substantial amount of surrounding Si atoms. The peaks at 485 and 600 cm^−1^ are attributed to the formation of four-membered (four oxygen atoms in the ring) and three-membered (three oxygen atoms in the ring) defects, respectively [26]. Very weak bands at 1,056 and 1,205 cm^−1^ (Figure 1) are similar to those associated with Si-O transverse and longitudinal optical modes [25,27].

In the graphene layers, the position, width, and intensity of the 2D peak depend on the number of graphene sheets in the sample [28]. In our case, the 2D band for the graphene monolayer (Figure 2) exhibits a single Lorentzian feature with a full width at half maximum (FWHM) of approximately 30 cm^−1^.

### Surface-enhanced scattering of Thy adsorbed on graphene-on-silica substrate

**Figure 3:**
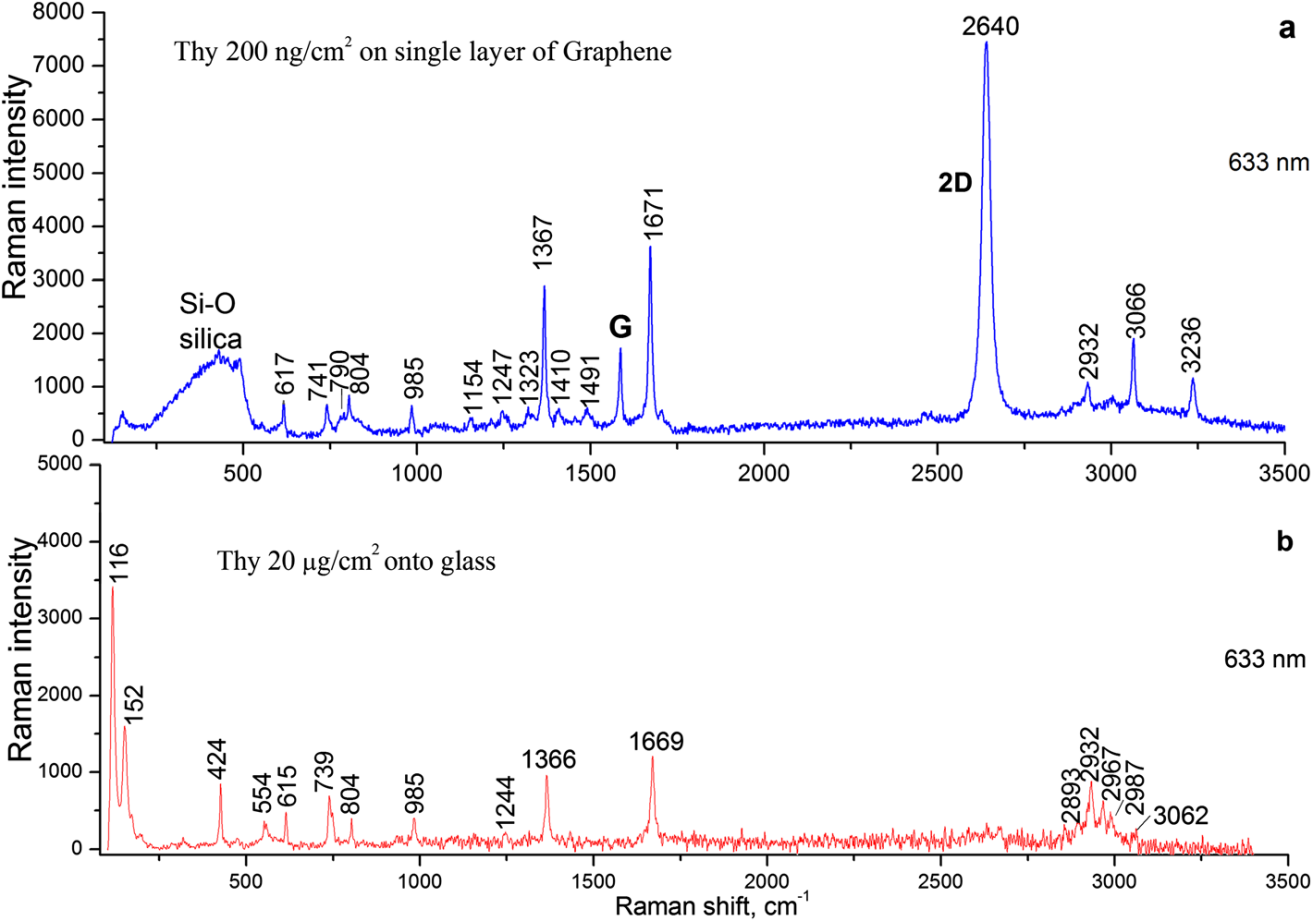
**SERS and Raman spectra.** SERS spectra for Thy adsorbed on single-layer graphene **(a)** and Raman spectra of Thy adsorbed on the glass surface **(b)**. Spectra are presented after baseline correction.

The Raman spectrum of Thy adsorbed on the graphene-on-silica substrate is shown in Figure 3a. One can observe that when Thy is adsorbed on single-layer graphene, the major intensive peaks in the Raman spectrum of Thy were blueshifted from 1,366 and 1,669 cm^−1^ (on glass, Figure 3b) to 1,367 and 1,671 cm^−1^ (on graphene, Figure 3a), respectively. By comparing Figure 3a,b, one can observe that the Thy Raman peak at 3,062 cm^−1^ shifts to 3,066 cm^−1^ and is strongly enhanced when Thy is adsorbed on graphene. It is worth noting also the change of the intensity of the Thy Raman peak at 2,932 cm^−1^.

In the Raman spectra of Thy adsorbed on single-layer graphene, one may clearly observe the characteristic G, 2D, and D peaks of graphene (Figure 3a). The adsorption of the Thy molecules onto the single-layer graphene results in the blueshift of the G peak and redshift of the 2D peak (see Table 1).

**Table 1:** **Band positions for single-layer graphene**

**Title of band**	**Single-layer graphene**	
	**Pristine Gr**	**Thy/Gr**
G	1,589	1,590
2D	2,644	2,640
*I* (2D)/*I* (G)	2.5	4.4
FWHM of G peak	30	27

The observed shifts of the G and 2D peaks indicate that deposition of Thy results in doping of graphene [29,30]. Specifically, the blueshift of the G band and redshift of 2D indicate *n*-doping of graphene by Thy molecules. FWHMs of the G band and the intensity ratio *I* (2D)/*I* (G) for bare and Thy-adsorbed graphene-on-silica substrates are presented in Table 2. One can see from Table 1 that adsorption of Thy decreases the FWHM of the G band for single-layer graphene. This experimental finding corresponds well to [30,31] where it has been shown that decrease of FWHM will saturate with the shift of the Fermi level. The ratio *I* (2D)/*I* (G) is also sensitive to the doping effects [32,33].

**Table 2:** **Assignment of the main Raman bands** (**cm** ^−**1**^) **observed for thymine**

**RAMAN spectra (** ***λ***_**ex**_ **= 633 nm)**		**Assignment in Thy** **[** 35 **-** 37 **]**
**Thy on glass**	**Thy on single-layer graphene**	
3,062	3,066	*ν*(C_6_H)
2,987	-	*ν*_as_(CH_3_)
2,967	-	*ν*_as_(CH_3_)
2,932	2,932	*ν*_s_(CH_3_)
2,893	-	*ν*_s_(CH_3_)
1,669	1,671	*ν*(C = O)
-	1,491	*δ*(N_1_-H), *ν*(ring),
-	1,410	*ν*(C_2_-N_3_), *δ*(N-H), *ν*(ring)
1,366	1,367	*δ*_s_(CH_3_), *δ*(N_3_-H)

*ν*, stretching; *δ*, deformation. All bands are assigned to Thy.

The GERS enhancement factor can be found by comparing the Raman signal obtained for Thy molecules deposited on glass (*I*_glass_) and graphene-on-silica (*I*_Gr_) substrates (see Figure 3) using the following equation:

$$ {g}_{\exp }=\frac{I_{\mathrm{Gr}}\left(\omega \right)\kern0.5em {m}_{\mathrm{glass}}\left(\mathrm{T}\mathrm{h}\mathrm{y}\right)}{I_{\mathrm{glass}}\left(\omega \right)\kern0.5em {m}_{\mathrm{Gr}}\left(\mathrm{T}\mathrm{h}\mathrm{y}\right)\kern0.5em } $$

where *m*_Gr_(Thy) and *m*_glass_(Thy) are the Thy surface densities on relevant substrates. Under our experimental conditions, we can well register already 200 ng/cm^2^ of Thy adsorbed on a single-layer graphene (Figure 3a, blue curve). However, when Thy surface density on the glass substrate is 200 ng/cm^2^, no Raman signal was observed at the same excitation intensity. For the registration of Raman spectra of Thy on glass, we increase surface densities of Thy up to 20 μg/cm^2^ (Figure 3b) to get a similar signal size to that on graphene. One can observe from Figure 3a that the GERS enhancement factor depends on the Thy molecular group and its interaction with the graphene surface. The maximum enhancement factor (*g*_exp_) of about 5 × 10^2^ was observed for the C(6)-H group and about 3 × 10^2^ for NH and C = O vibrations of Thy molecules adsorbed on single-layer graphene.

The assignments of Raman shift for thymine adsorbed on graphene and glass substrate are listed in Table 2.

The band observed for Thy adsorbed on single-layer graphene in Figure 3a (blue curve) at 617 cm^−1^ is attributed to the wag of N-H in the structure of Thy, 741 cm^−1^ is attributed to ring breathing and coupled to the out-plane wag of N-H, 804 cm^−1^ is attributed to the wag of C-H on C = C, 985 cm^−1^ is attributed to ring breathing coupled to in-plane -CH_3_ asymmetric stretching, 1,367 cm^−1^ is attributed to N-H and C-H in-plane bending, and 1,671 cm^−1^ is attributed to C = O stretching and coupled to N-H and C-H asymmetric bending. The bands of 1,410 and 1,491 cm^−1^ were also the characteristic peaks of GERS for ring modes of Thy, not visible on glass. The bands centered at 116 and 152 cm^−1^ in the Raman spectra deposited on the glass substrate indicate the presence of three-dimensional micro-crystallites of Thy due to the lower sticking probability on glass compared to graphene. These bands do not appear in the Raman spectra of Thy on the graphene-on-silica substrate. The microscopy images also show that Thy adsorbed on graphene seems to form flat flakes - the same observation we had in our previous work [10] for Thy adsorbed on graphene oxide. Due to the presence of big three-dimensional micro-crystallites of Thy on glass, the broad band at 250 to 600 cm^−1^ which is associated with the molecular vibrations in the substrate is absent in Figure 3b, while in the case of Thy on the graphene-on-silica substrate (Figure 3a), the band at 250 to 600 cm^−1^ is available due to the very small thickness of flakes of Thy.

The band at 3,066 cm^−1^ in GERS spectra of Thy, which is attributed to the aromatic C(6)-H stretching vibration, is strongly enhanced, while the band at 2,932 cm^−1^, which is assigned to the symmetric CH_3_ stretching vibration, is decreased (Figure 3). The possible orientation of Thy with respect to the graphene surface and binding sites between adsorbed molecules and graphene needs additional investigations and discussions.

### Graphene-enhanced coherent anti-Stokes Raman scattering of thymine

**Figure 4:**
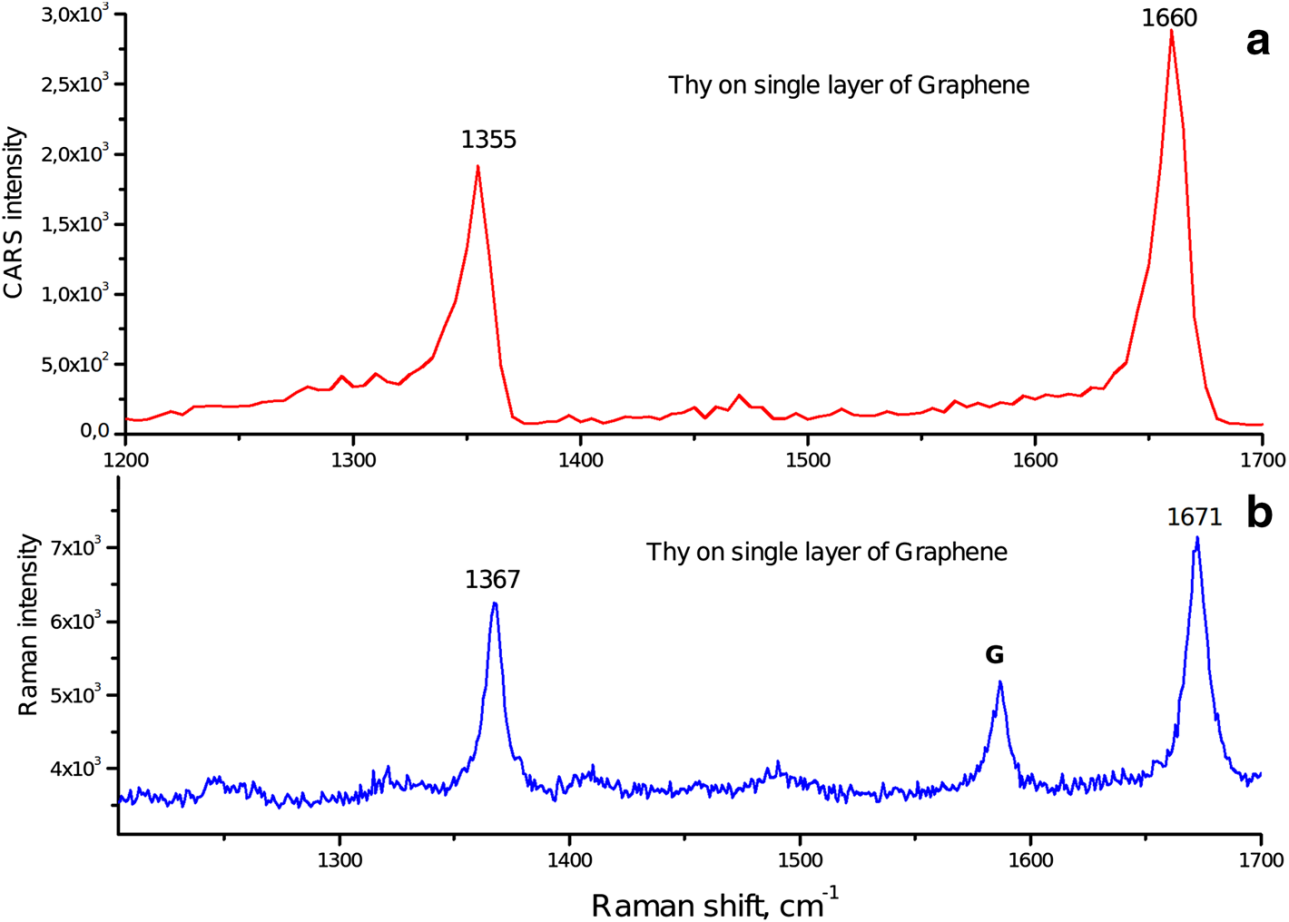
**Comparison CARS spectra (a) with GERS spectra (b) of Thy on single-layer graphene.**

**Figure 5:**
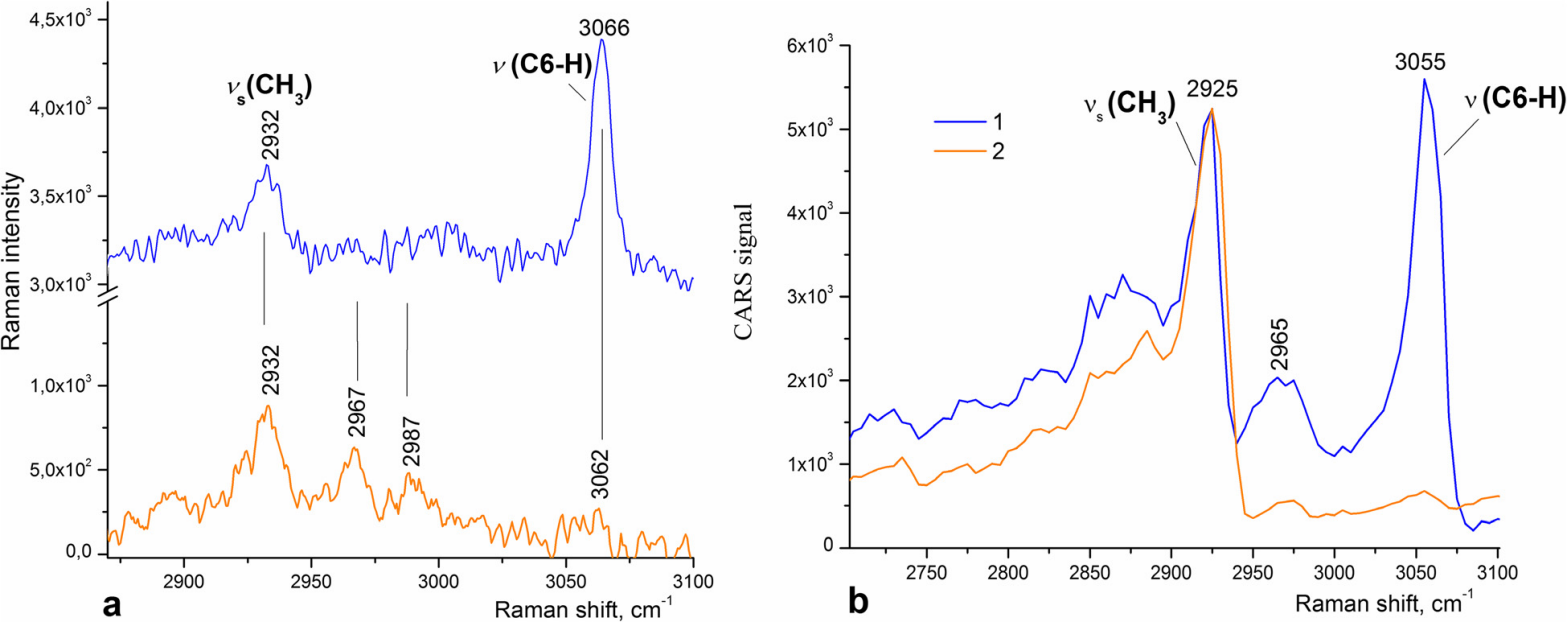
**Raman and CARS spectra. (a)** Raman spectra of Thy adsorbed on single-layer graphene (blue) and glass (yellow). **(b)** CARS spectra of Thy adsorbed on single-layer graphene (blue) and on glass (yellow). Major Raman lines of Thy molecules are shown.

The CARS measurements for the Thy adsorbed on different substrates were carried out in spectral ranges of 1,200 to 1,700 cm^−1^ and 2,500 to 3,400 cm^−1^. CARS and Raman spectra of Thy are quite similar: main bands are about 10 cm^−1^ shifted to the short wavenumber region (Figures 4 and 5). The low-energy shift of the band in CARS spectra is an intrinsic feature of this technique caused by mixing of resonance (vibrational) response with non-resonance contribution.

Another relevant feature of the CARS method is the quadratic CARS signal dependence on concentration of matter. For this reason, the CARS spectra contain strong bands of Thy and the vanished G band of graphene (Figure 4a). Additionally, the coherence origin of CARS may have an effect on the signal weakening from graphene.

One can observe from Figure 5 that the Thy adsorption on graphene results in the redistribution of the intensity of the Thy Raman bands in the 2,700- to 3,100-cm^−1^ range and enhancement of the ν(C6-H) mode at 3,055 cm^−1^ in the CARS spectrum. The aromatic C(6)-H stretching band of Thy in Raman spectra is observed at 3,062 cm^−1^, and in CARS spectra, this band shifts to 3,055 cm^−1^. In addition, we have to indicate that this band is indistinguishable for samples prepared on glass substrates (Figure 5) in both Raman and CARS spectra.

The important difference between the CARS spectrum of Thy adsorbed on glass and that on graphene-on-silica substrates (yellow and blue curves in Figure 5b) must be noted: in the CARS spectrum of Thy adsorbed on the graphene-on-silica substrate, the band near 2,965 cm^−1^ is stronger than that of Thy adsorbed on glass. We have recently observed enhancement for the CARS signal of Thy adsorbed on graphene oxide [10] where the broadening of the CARS band at 2,700 to 3,100 cm^−1^ originated from the electron-phonon and phonon-phonon coupling.

**Figure 6:**
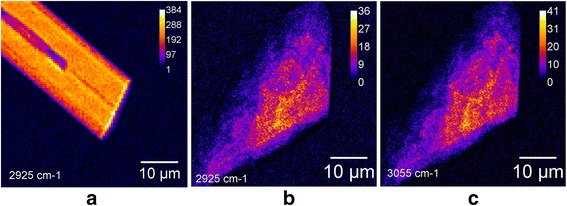
**CARS images of Thy adsorbed on different substrates. (a)** On glass and **(b,c)** on graphene monolayer.

Figure 6 shows CARS microscopy images in a 50 × 50-μm area of Thy adsorbed on glass and graphene-on-silica substrates. One can observe from Figure 6a,b that at 2,925 cm^−1^, both substrates provide high-contrast images of adsorbed Thy molecules. In contrast, at 3,055 cm^−1^, the Thy molecules adsorbed on graphene provide a strong CARS signal over the sample surface (Figure 6c), while Thy molecules adsorbed on glass give no CARS signal (not shown). The Thy molecules on glass form crystals with an average size of several tens of micrometers. We can see that adsorbed Thy molecules deposited on the graphene sheet form complex Thy/graphene as flat flakes with a lateral size up to 30 μm. The image at 3,055 cm^−1^ illustrates enhancement of the CARS signal from Thy molecules on the graphene monolayer. The CARS signal intensity of both 2,925 and 3,055 cm^−1^ bands of the Thy adsorbed on graphene dramatically reduced with the distance from the graphene surface.

The performed Raman and CARS measurements reveal that signal enhancement manifests itself similarly in both linear and nonlinear experiments. This enhancement may originate from charge transfer between the Thy molecules and the graphene surface that results in increase of the molecular polarizability. The experimentally observed doping-related shift of the G and 2D Raman peaks of graphene (see Table 1) supports the importance of the charge transfer. However, the resonant interaction of exciting light with electronic states of the graphene sheets and the increase of the local field at the defects [34] and edges of the graphene layers can also contribute to the observed enhancement. Since the plasmon resonance for graphene is usually situated in the THz range [5,6], we believe that the observed GERS is due to the formation of the charge transfer between the Thy molecules and the graphene sheet.

## Conclusions

GERS was studied for the Thy adsorbed on single-layer graphene. The observed enhancement of the Raman signal of more than 100 times was observed for C(6)-H, NH, and C = O vibrations of Thy molecules adsorbed on single-layer graphene and was accompanied with a minor shift of these Thy bands. The highest enhancement in GERS effect was observed for ring modes of thymine. The CARS spectra of Thy adsorbed on single-layer graphene are in accord with GERS of Thy and demonstrated redistribution of the intensity of the Thy Raman bands in the 2,800- to 3,100-cm^−1^ range and enhancement of the ν(C6-H) mode at 3,055 cm^−1^ in the CARS and at 3,066 cm^−1^ in Raman, respectively. The change in GERS compared to ordinary Raman strongly depends on the vibrational mode of adsorbed molecules and thus can provide an insight into the chemical mechanism.

## Abbreviations

CARS: coherent anti-Stokes Raman scattering
GECARS: graphene-enhanced coherent anti-Stokes Raman scattering
GERS: graphene-enhanced Raman scattering
SERS: surface-enhanced Raman scattering
Thy: thymine
